# Progression of the estimated glomerular filtration rate in asphyxiated neonates undergoing therapeutic hypothermia during the first 10 days of life

**DOI:** 10.1007/s00467-025-06957-1

**Published:** 2025-09-18

**Authors:** Karel Allegaert, Julia Macente, Djalila Mekahli, John van den Anker, Pieter Annaert, Anne Smits

**Affiliations:** 1 Clinical Pharmacology and Pharmacotherapy, Department of Pharmaceutical and Pharmacological Sciences, KU Leuven, Herestraat 49, P.O. Box 611, Leuven, 3000 Belgium; 2 Department of Development and Regeneration, KU Leuven, Leuven, 3000 Belgium; 3 Department of Hospital Pharmacy, Erasmus University Medical Center, Rotterdam, CA 3000 The Netherlands; 4 Drug Delivery and Disposition, Department of Pharmaceutical and Pharmacological Sciences, KU Leuven, Leuven, 3000 Belgium; 5 PKD Research Group, Laboratory of Ion Channel Research, Department of Cellular and Molecular Medicine, KU Leuven, Leuven, Belgium; 6 Department of Pediatric Nephrology, University Hospitals Leuven, Leuven, 3000 Belgium; 7 Center for Translational Research, Children’s National Hospital, Washington, DC 20010 USA; 8 BioNotus GCV, Niel, Antwerp, 2845 Belgium; 9 Neonatal Intensive Care Unit, University Hospitals Leuven, Leuven, 3000 Belgium

**Keywords:** Estimated glomerular filtration rate, Newborn, Asphyxia, Acute kidney injury, Therapeutic hypothermia

## Abstract

**Background:**

Serum creatinine (Scr) centile values were recently described in a cohort of 1136 (near)-term neonates that underwent therapeutic hypothermia (TH) because of moderate to severe hypoxic-ischemic encephalopathy. Recent methodological progress enables conversion of these Scr centiles to estimated glomerular filtration rate (eGFR) values.

**Methods:**

Scr centiles in the TH dataset during the first 10 days of life were converted to eGFR values, using the Schwartz formula, with the Smeets k-value (0.31) and fixed body length (50 cm) to generate postnatal reference eGFR values, centiles, and an equation for median eGFRs. These findings were compared to published eGFR data in term controls.

**Results:**

A polynomial function was estimated: $\mathrm{eGFR} \left(\frac{\mathrm{mL}}{\mathrm{min}}\bullet 1.73{ \mathrm{m}}^{2}\right)=9.1667+7.1173 x -0.3439{x}^{2},(x=\mathrm{days})$ for eGFR in TH neonates. The median eGFR increases 2- to threefold over the first week (day 1: 16.1; day 2: 19.4; day 7: 41.2 mL/min∙1.73 m^2^), while the polynomial function does not fully reflect the interindividual variability in eGFR values (intra-day variability is also 2- to threefold). Patterns in acute kidney injury (AKI) TH cases differ significantly from non-AKI TH cases. Based on pooling of published eGFR data, this was compared to a function in healthy term neonates: $\mathrm{eGFR} \left(\frac{\mathrm{mL}}{\mathrm{min}}\bullet 1.73 {\mathrm{m}}^{2}\right)=14.2167+6.7644 x-0.3901{x}^{2}(x=\mathrm{days})$ (day 1: 20; day 2: 26; day 7: 42 mL/min/1.73 m^2^).

**Conclusions:**

Based on a pooled dataset in TH cases, we converted Scr centiles to eGFR centiles. Based on median values, this resulted in a polynomial function in TH cases, compared to healthy term neonates. This eGFR function enables precision pharmacotherapy for GFR-cleared drugs in this vulnerable population.

**Graphical abstract:**

**Figure:**
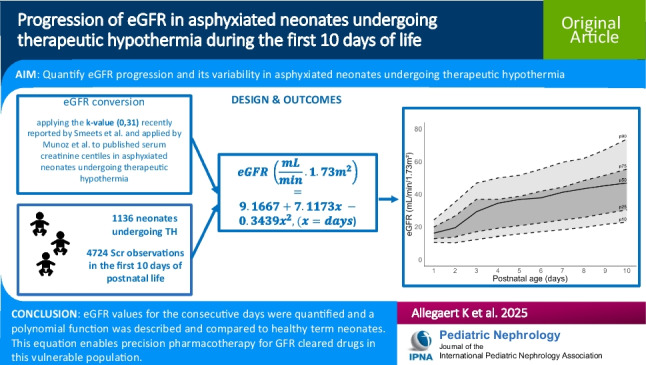
A higher resolution version of the Graphical abstract is available as Supplementary information

**Supplementary Information:**

The online version contains supplementary material available at 10.1007/s00467-025-06957-1.

## Introduction

Newborns are vulnerable to kidney impairment or acute kidney injury (AKI), with a reported AKI incidence of 20–30% in the Assessment of Worldwide AKI Epidemiology in Neonates (AWAKEN) study. AKI is associated with a higher risk of mortality and length of stay [1]. This vulnerability is mainly due to their very fast postnatal maturational changes in glomerular filtration rate (GFR). These changes are almost exclusively driven by major increases in renal and glomerular perfusion after birth. Consequently, the neonatal kidney is very sensitive to disturbed vasoreactivity. The fast maturational changes in GFR have recently been re-illustrated in this journal by Muñoz et al. [2]. The authors observed a twofold increase in estimated GFR (eGFR) over the first week of postnatal life in term neonates, with an increase in mean eGFR from 19.5 to 41.8 mL/min∙1.73 m^2^. These findings confirm measured GFR (mGFR) patterns described by Wu et al., and by Smeets et al. in term neonates [3, 4].

Wu et al. pooled inulin observations in the first 90 days of life. The authors concluded that mGFR maturation was indeed fast and associated with a postnatal-related maturation that was dependent on the gestational age [3]. Smeets et al. specifically focused on term-born neonates and performed an individual participant data meta-analysis based on reported mGFR values, not limited to inulin [4]. These authors concluded that GFR doubles in the first 5 days of life, with a subsequent more gradual increase. Interestingly, these authors also estimated an updated Schwartz equation coefficient (*k*, 0.31), as also applied by Muñoz to convert Scr to eGFR values [eGFR (mL/min∙1.73 m^2^) = 0.31 × height (cm)/Scr (mg/dL)] [2, 4].

Interestingly, these methodological advances enable further exploration of disease-related eGFR patterns over postnatal age in specific subpopulations of term neonates, such as neonates during or following therapeutic hypothermia (TH) after moderate to severe hypoxic-ischemic encephalopathy, a population known to commonly suffer from kidney impairment in neonatal life as part of a multi-organ clinical syndrome [1, 5].

We previously reported on Scr trends, their variability (centile values), and differences compared to a reference group of (near)-term cases admitted to the neonatal intensive care unit (NICU) [5]. However, to better understand the differences in GFR-related drug clearance during postnatal age, for example, or to better target the potential benefit of methylxanthines to prevent kidney impairment in this scenario, a conversion to eGFR values in TH cases and development of eGFR equations in TH cases, compared to controls, is valuable.

We therefore aimed to calculate the eGFR values based on published centile Scr values over the first 10 days of postnatal life in asphyxiated neonates undergoing TH [5]. We deliberately reported values as centiles to reflect the extensive interpatient variability. Within this TH cohort, we compared findings in TH neonates who developed AKI versus TH neonates who did not develop AKI. As our pooled dataset had no information on urine output, the AKI definition was based on Scr trends, using the KDIGO definition (SCr↑ ≥ 0.3 mg/dL within 48 h, or Scr ↑ ≥ 1.5-fold versus the lowest prior Scr within 7 days) [5, 6].

We subsequently compared mean eGFR values and postnatal trends in TH cases to the estimates reported by Muñoz et al., Wu et al., and Smeets et al. in term controls, and generated eGFR equations in both TH cases and term controls [2–4].

## Methods

### Datasets in TH cases

Datasets from 7 different TH-treated cohorts with 4724 Scr values in 1136 TH neonates were recently pooled, of whom 132 (11.6%) were classified as having AKI [5, 6]. To facilitate pooling, variables collected were restricted to birth weight, gestational age, neonatal survival, and the Scr values (no data on sex or body length). Postnatal day 1 was defined as the date of delivery [5]. In the original paper, we reported on centile (p10, p25, p50, p75, p90, and p95) Scr values from day 1 up to day 10.

These centile values were used to calculate the eGFR [eGFR (mL/min.1.73 m^2^) = 0.31 × height (cm)/Scr (mg/dL)], as reported by Smeets et al. [4], and recently applied by Muñoz et al. [2]. For ethics-related aspects, or additional methodological details or cohort characteristics, we refer to the original paper [5].

### eGFR centile calculation in TH cases

Centile eGFR values were calculated as suggested by Smeets et al. and as recently reported by Muñoz et al. [2, 4]. Since body length is only rarely collected and is notoriously known to show inter- and intra-observer variability in newborns, and since all cases were (near)term neonates, we arbitrarily used a fixed value (50 cm), extrapolated from the median birth weight (3350 g) of our dataset [5]. We hereby used the modified Schwartz formula, with the *k* value suggested by Smeets (*k* = 0.31) [4].

Finally, we are aware of a median mannitol clearance estimate in neonates undergoing TH and have recalculated this value (0.15 L/h, mean weight 3.25 kg [7]) to a measured GFR value (17 mL/min∙1.73 m^2^, on day 2) to enable comparison to the eGFR on day 2 in TH neonates [5, 7]. While this study reports on 77 mannitol measurements in 17 asphyxiated neonates, the study design (intermittent administration, at random sampling over the first 3 days of postnatal life), did not allow us to calculate mGFR values besides the reported median mannitol clearance value [7].

### eGFR or mGFR values in controls

Postnatal day-specific eGFR values as reported for term neonates in the publications of Muñoz et al. (critical care), Wu et al. (mixed), and Smeets et al. (healthy) were extracted from the source documents [2–4]. For the Muñoz et al. and Smeets et al. papers, this was based on the values reported in the table(s) of their publications [2, 4]. For the Wu et al. paper, data were extracted from their Fig. 4 (gestational age 40.2 weeks, birth weight 3600 g), using extraction software (WebPlotDigitizer, version 5) [3]. For similarity, day 1 was defined as the day of delivery for all cohorts.

### Estimation of a postnatal eGFR equation using regression

To estimate a postnatal eGFR equation in both TH cases and controls based on the 50th centile values, a regression-based approach was developed. Initially, we explored several models, including linear, logarithmic, exponential, and polynomial regression to determine the better model that fit the data. To evaluate the fit of each model, we used the Akaike information criterion (AIC) and the coefficient of determination (*R*^2^). These statistical metrics were used to assess the relative goodness of fit, with lower AIC values and higher *R*^2^ values indicating a better model.

## Results

### eGFR in TH cases

**Fig. 1:**
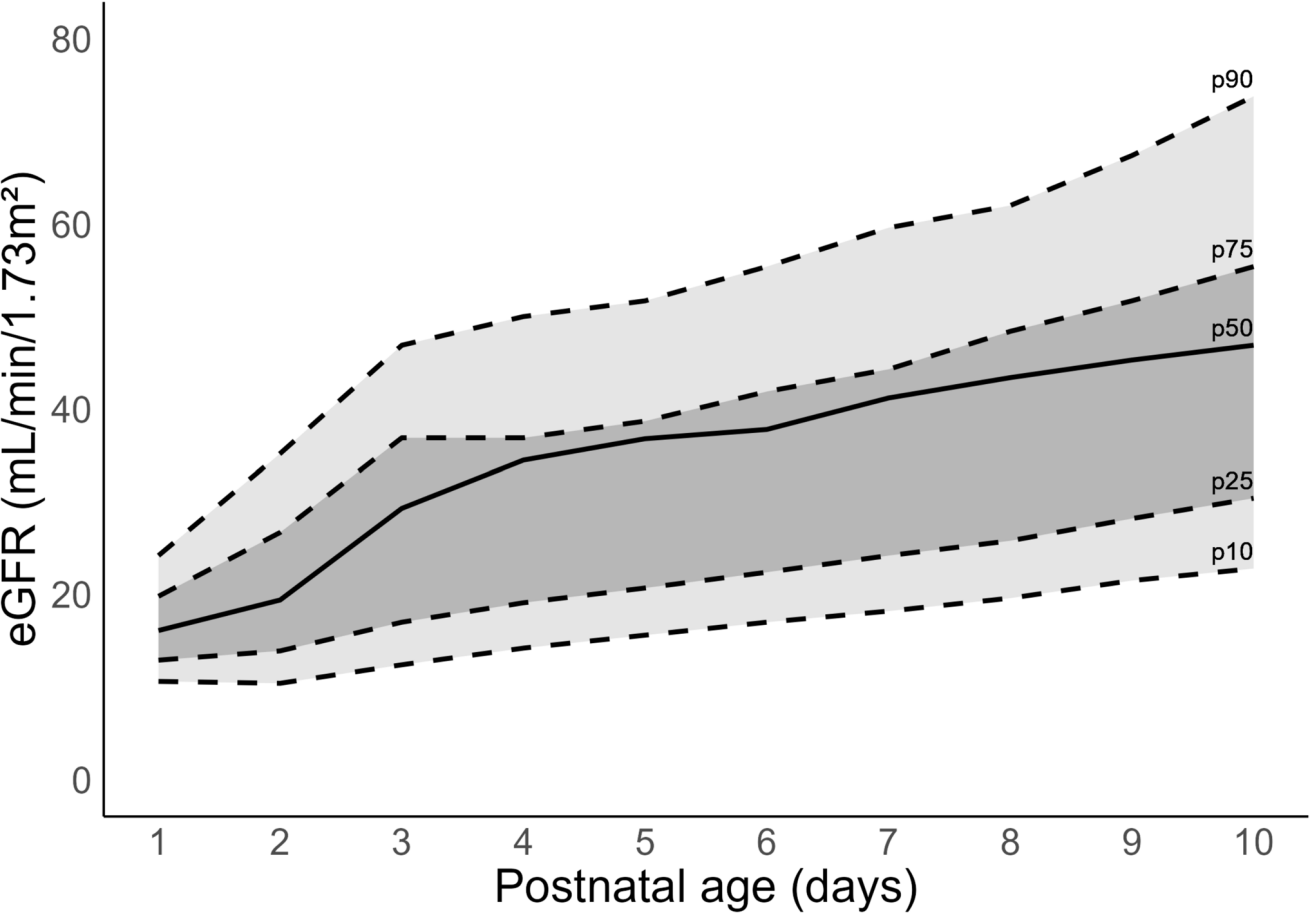
Estimated GFR (eGFR) in the first 10 days of asphyxiated neonates undergoing therapeutic hypothermia. The solid black line represents the estimated median eGFR, the darker gray represents the interval between p25 and p75, while the light gray represents the 10th and 90th percentiles of eGFR in this population [4]

The eGFR values as calculated (median weight 3350 g, length 50 cm) for the consecutive days are provided in Table 1. In Fig. 1, we plotted the eGFR values (p10-p25-p50-p75-p90) for the TH cohort during postnatal age. Figure 1 clearly reflects the quantitative increase in eGFR over postnatal age, as well as the extensive in-between variability in eGFR values (centiles) over postnatal age. We observed a 2- to threefold increase in eGFR over the first week of life, while the extensive range in eGFR centiles for a given postnatal age (also 2- to threefold differences between the 10th and 90th centile), illustrate the clinically relevant between-patient variability in kidney impairment and eGFR.

**Table 1:** Centiles (10–90th) of eGFR (mL/min∙1.73 m^2^) over postnatal age in 1136 asphyxiated neonates who underwent therapeutic hypothermia, based on 4724 serum creatinine observations [5]

Centile	Day 1	Day 2	Day 3	Day 4	Day 5	Day 6	Day 7	Day 8	Day 9	Day 10
P90	24.2	35.2	46.9	50	51.7	55.4	59.6	62	67.4	73.8
P75	19.8	26.7	34.4	36.9	38.7	41.9	44.3	48.4	51.7	55.4
p50	16.1	19.4	29.3	34.5	36.8	37.8	41.2	43.4	45.3	46.9
P25	12.9	13.9	17	19.1	20.7	22.4	24.2	25.8	28.2	30.4
P10	10.6	10.4	12.4	14.2	15.6	17	18.2	19.6	21.5	22.8
p5	9.5	8.8	10.3	11.8	13.1	14.5	15.5	18.2	18.3	19.4

To further emphasize this extensive inter-individual variability between TH cases, we also calculated the median eGFR patterns over postnatal age in neonates who either developed or did not develop AKI (Table 2). Comparing both subgroups of TH cases, Table 2 further reflects clinically significant differences and variability in eGFR over postnatal age. Intriguingly, there is an increase in eGFR over postnatal age in both groups.

**Table 2:** Mean eGFR (mL/min∙1.73 m^2^) over postnatal age in 1136 asphyxiated neonates who underwent therapeutic hypothermia, of whom 132 (11.6%) were classified as having AKI [5]

	Day 1	Day 2	Day 3	Day 4	Day 5	Day 6	Day 7	Day 8	Day 9	Day 10
AKI	13.7	10.6	10.4	11.1	12.1	13.5	13.9	18.9	21.2	25
No AKI	16.3	20.4	26.7	29.8	31.6	33.7	36.0	38.7	41.9	44.3

### mGFR or eGFR in controls, and comparison to eGFR patterns in TH cases

Median mGFR estimates as reported by Smeets et al. [4], and eGFR estimates as reported by Wu et al. [3], and Muñoz et al. [2] are provided in Table 3, combined with the 10th, 50th, and 90th centile eGFR values as observed in the TH cohort.

**Table 3:** Centiles (10th–50th–90th) of eGFR (mL/min∙1.73m^2^) over postnatal age in 1136 asphyxiated neonates who underwent therapeutic hypothermia (TH), compared to mean eGFR values as reported in term controls [2–5]

Cohort	Day 1	Day 2	Day 3	Day 4	Day 5	Day 6	Day 7	Day 8	Day 9	Day 10
TH cases, 90^th^	24.1	35.2	46.9	50	51.7	55.4	59.6	62	67.4	73.8
TH cases, 50^th^	16.1	19.4	29.3	34.5	36.8	37.8	41.2	43.4	45.3	46.9
TH cases, 10^th^	10.6	10.4	12.4	14.2	15.6	17	18.2	19.6	21.5	22.8
Smeets et al. [4]	20	26	32	35	39	41	42	42	43	44
Wu et al. [3]	20	25	29	34	37	38	41	43	45	47
Munoz et al. [2]	20	22	n.a.	33	n.a.	n.a.	42	n.a.	n.a.	n.a.

### Estimation of postnatal egfr equations

Applying the regression approach to the 50th centile eGFR data, a function was estimated: $\mathrm{eGFR} \left(\frac{\mathrm{mL}}{\mathrm{min}}.1.73{ \mathrm{m}}^{2}\right)=9.1667+7.1173 x-0.3439{x}^{2} (x=\mathrm{postnatal days})$ for eGFR in neonates undergoing or following TH. The mannitol-derived median mGFR in TH cases on day 2 was 17 mL/min∙1.73 m^2^ [7].

**Fig. 2:**
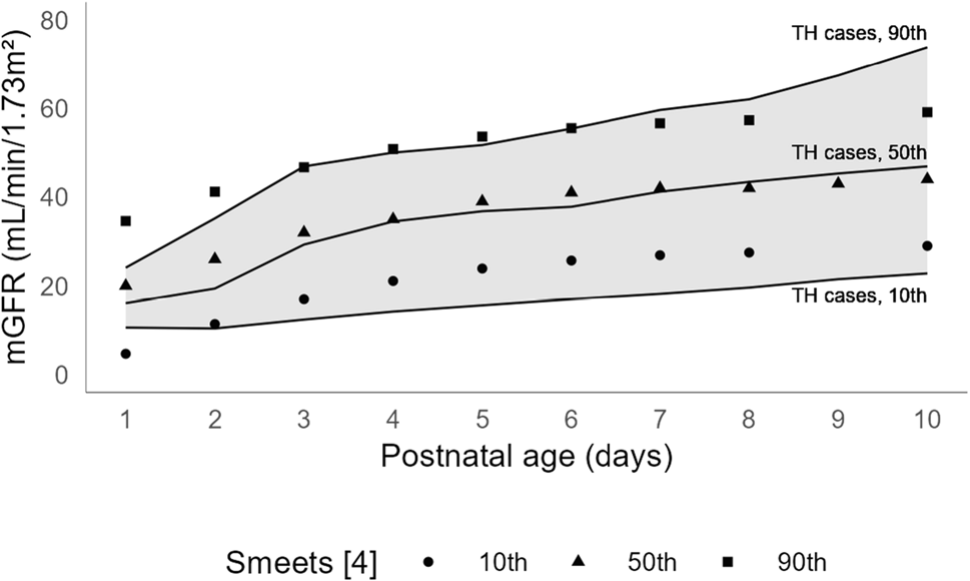
Estimated GFR (eGFR) in the first 10 days of asphyxiated neonates undergoing therapeutic hypothermia (TH), compared to observed measured GFR (mGFR) data from Smeets et al. in healthy control neonates. Solid lines represent the TH cases at p90, p50, and p10 [4, 5]

Based on the median values reported by Smeets et al. in controls [4], an equation for eGFR was estimated: $\mathrm{eGFR} \left(\frac{\mathrm{mL}}{\mathrm{min}}.1.73{ \mathrm{m}}^{2}\right)=14.2167+6.7644 x-0.3901{x}^{2} (x=\text{postnatal days})$. In Fig. 2, we plotted the eGFR values for the TH combined with the mGFR values previously reported by Smeets et al. Table 3 and Fig. 2 hereby illustrate the quantitative differences in the postnatal pattern of GFR estimates in TH compared to controls over postnatal age.

## Discussion

The newly generated reference eGFR values and eGFR equation in this specific neonatal subpopulation were compared to a reference population, as this provides better insights into eGFR estimates, and into kidney impairment-related pathophysiological patterns over postnatal age [8]. The median eGFR estimate on day 2 in TH cases is also very similar to the median mGFR estimate (mannitol) (19.4 versus 17 mL/min∙1.73 m^2^) reported [7]. Comparing the postnatal pattern of eGFR between TH cases and controls, Fig. 2 shows a median lower eGFR over the first 3 days of postnatal age, with a portion of TH cases that display (10th centiles) a blunted postnatal recovery pattern for eGFR. In this way, reference eGFR values hold the promise to further support clinical research practices and facilitate secondary analysis of the impact of interventions on kidney-related outcomes.

Such data can be applied to develop tailored dosing of medicines cleared by GFR, to consider targeted secondary kidney impairment prevention in TH cases (e.g., methylxanthines), or to develop targeted post-discharge nephrological follow-up in ‘outliers’ [9]. Similar approaches could be considered for other subpopulations of term neonates, e.g., neonates undergoing cardiac surgery or extra-corporeal membrane oxygenation [1].

When considering the application of these eGFR estimates and equations, it is important to be aware that TH itself reduces kidney impairment, since AKI incidence (relative risk 0.81, 95% CI 0.67–0.98) is lower in TH cases when compared with neonates with moderate to severe hypoxic-ischemic encephalopathy not undergoing TH [10]. Tailored dosing regimen development with the current data should therefore focus on TH-treated cases and should not be extrapolated to non-TH-treated, moderate to severe asphyxia cases.

Related to targeted prevention strategies, it has been reported that restrictive fluid intake (< 60 mL/kg) was associated with a higher risk of developing AKI (12% versus 5%, *p* = 0.02) in this population, while aminophylline (loading dose 5 mg/kg, followed by 1.8 mg/kg.q6 h) was associated with a lower risk of developing AKI (higher urinary output, and a decline in Scr values) [11, 12]. With the current new data, capturing the inter-patient variability in eGFR values, a subgroup of neonates that is more likely to benefit from the administration of methylxanthines can be identified, applying centile values, for example. From a nephrological perspective, it is important to realize that the available information on pediatric kidney post-discharge outcome after TH-treated hypoxic-ischemic encephalopathy currently remains very scant and limited [13].

Finally, these equations can be used to better capture eGFR patterns in physiologically based pharmacokinetic (PBPK) models to predict drug exposure over postnatal age in this specific population [14].

While equations based on median values for specific populations are very valuable, it is important to stress the limitations of the current effort. Median trends insufficiently capture inter-patient variability, so that population trends do not reflect the individual patterns well. This is reflected in Table 2 and Fig. 1, as the postnatal recovery patterns are different between AKI and non-AKI cases, while Fig. 1 shows an extensive range in eGFR centiles. Furthermore, new data are warranted to either confirm or modulate the current eGFR estimates. Finally, we have arbitrarily fixed the body length to 50 cm. While this is a reasonable option, imputation based on weight and gestational age is an alternative, as done by Smeets et al. However, this approach did not result in differences in their eGFR estimates in an additional sensitivity analysis, suggesting that the currently applied pragmatic approach is fair and reasonable [4].

## Conclusions

Based on a pooled dataset of 1136 asphyxiated neonates undergoing TH in different units, we have converted Scr centiles to eGFR values, applying the recently described approach of Smeets et al. and Muñoz et al. and have compared these to reference values. This eGFR function enables precision pharmacotherapy of GFR-cleared drugs in this vulnerable population. Furthermore, this opens the way to better explore the impact of targeted interventions on kidney (e.g., methylxanthines)-related outcomes in this specific population and may identify cases that warrant long-term kidney follow-up.

## Supplementary Information

Below is the link to the electronic supplementary material.

Graphical abstract (PDF 247 KB)

## Data Availability

Data that support the paper are already available in the public domain.
