# Correction: Progression of the estimated glomerular filtration rate in asphyxiated neonates undergoing therapeutic hypothermia during the first 10 days of life

**DOI:** 10.1007/s00467-026-07474-5

**Published:** 2026-08-03

**Authors:** Karel Allegaert, Julia Macente, Djalila Mekahli, John van den Anker, Pieter Annaert, Anne Smits

**Affiliations:** 1 Clinical Pharmacology and Pharmacotherapy, Department of Pharmaceutical and Pharmacological Sciences, KU Leuven, Herestraat 49, P.O. Box 611, 3000 Leuven, Belgium; 2 Department of Development and Regeneration, KU Leuven, 3000 Leuven, Belgium; 3 Department of Hospital Pharmacy, Erasmus University Medical Center, Rotterdam, CA 3000 The Netherlands; 4 Drug Delivery and Disposition, Department of Pharmaceutical and Pharmacological Sciences, KU Leuven, 3000 Leuven, Belgium; 5 PKD Research Group, Laboratory of Ion Channel Research, Department of Cellular and Molecular Medicine, KU Leuven, Leuven, Belgium; 6 Department of Pediatric Nephrology, University Hospitals Leuven, 3000 Leuven, Belgium; 7 Center for Translational Research, Children’s National Hospital, Washington, DC 20010 USA; 8 BioNotus GCV, Niel, 2845 Antwerp, Belgium; 9 Neonatal Intensive Care Unit, University Hospitals Leuven, 3000 Leuven, Belgium

**Correction: Pediatric Nephrology (2025) 41:233–238.**

**https://doi.org/10.1007/s00467-025-06957-1**

The original online version of this article was revised to correct formulas that were incorrectly reported on the first page (abstract), on page 3 and in the graphical abstract.

The formula.

$$\mathrm{e}\mathrm{G}\mathrm{F}\mathrm{R}\;\left(\mathrm{m}\mathrm{L}/\mathrm{m}\mathrm{i}\mathrm{n} 1.73 {\mathrm{m}}^{2}\right)=9.1667+7.1173-0.3439{\mathrm{x}}^{2}\;\left(\mathrm{x}=\mathrm{d}\mathrm{a}\mathrm{y}\mathrm{s}\right)$$

has been corrected to.

$$\mathrm{e}\mathrm{G}\mathrm{F}\mathrm{R}\;\left(\mathrm{m}\mathrm{L}/\mathrm{m}\mathrm{i}\mathrm{n} 1.73 {\mathrm{m}}^{2}\right)=9.1667+7.1173\;\mathrm{x}-0.3439{\mathrm{x}}^{2}\left(\mathrm{x}=\mathrm{d}\mathrm{a}\mathrm{y}\mathrm{s}\right).$$

In addition, the formula.

$$\mathrm{e}\mathrm{G}\mathrm{F}\mathrm{R}\;\left(\mathrm{m}\mathrm{L}/\mathrm{m}\mathrm{i}\mathrm{n} 1.73 {\mathrm{m}}^{2}\right)=14.2167+6.7644-0.3901{\mathrm{x}}^{2}\;\left(\mathrm{x}=\mathrm{d}\mathrm{a}\mathrm{y}\mathrm{s}\right)$$

has been corrected to.

$$\mathrm{e}\mathrm{G}\mathrm{F}\mathrm{R}\left(\mathrm{m}\mathrm{L}/\mathrm{m}\mathrm{i}\mathrm{n} 1.73 {\mathrm{m}}^{2}\right)=14.2167+6.7644\mathrm{x}-0.3901{\mathrm{x}}^{2}\left(\mathrm{x}=\mathrm{d}\mathrm{a}\mathrm{y}\mathrm{s}\right).$$

The formula has also been corrected in the graphical abstract reproduced here:

**Figure:**
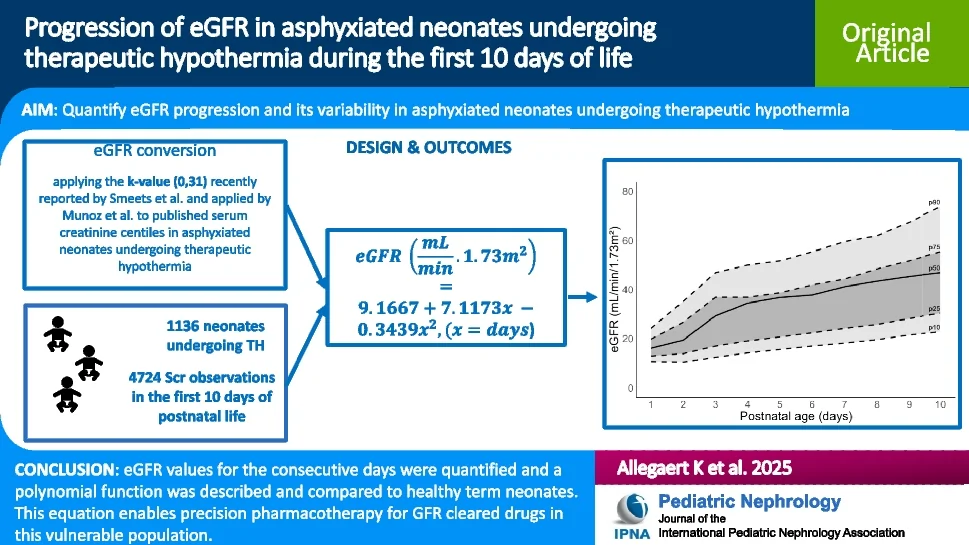

The original article has been corrected.

