# Supplementary material for: Progression of the estimated glomerular filtration rate in asphyxiated neonates undergoing therapeutic hypothermia during the first 10 days of life
Source: Pediatr Nephrol. 2025 Sep 18;41(1):233–8. doi: 10.1007/s00467-025-06957-1 (PMC12686099; doi:10.1007/s00467-025-06957-1)
Supplement: Supplementary file 1 — Graphical abstract (PDF 247 KB) [file 467_2025_6957_MOESM1_ESM.pdf]

Progression of eGFR in asphyxiated neonates undergoing therapeutic hypothermia during the first 10 days of life

Original Article

AIM: Quantify eGFR progression and its variability in asphyxiated neonates undergoing therapeutic hypothermia

eGFR conversion

applying the **k-value (0,31)** recently reported by Smeets et al. and applied by Munoz et al. to published serum creatinine centiles in asphyxiated neonates undergoing therapeutic hypothermia

1136 neonates undergoing TH

4724 Scr observations in the first 10 days of postnatal life

DESIGN & OUTCOMES

$$eGFR\left(\frac{mL}{min}.1.73m^2\right) = 9.1667 + 7.1173x - 0.3439x^2, (x = days)$$

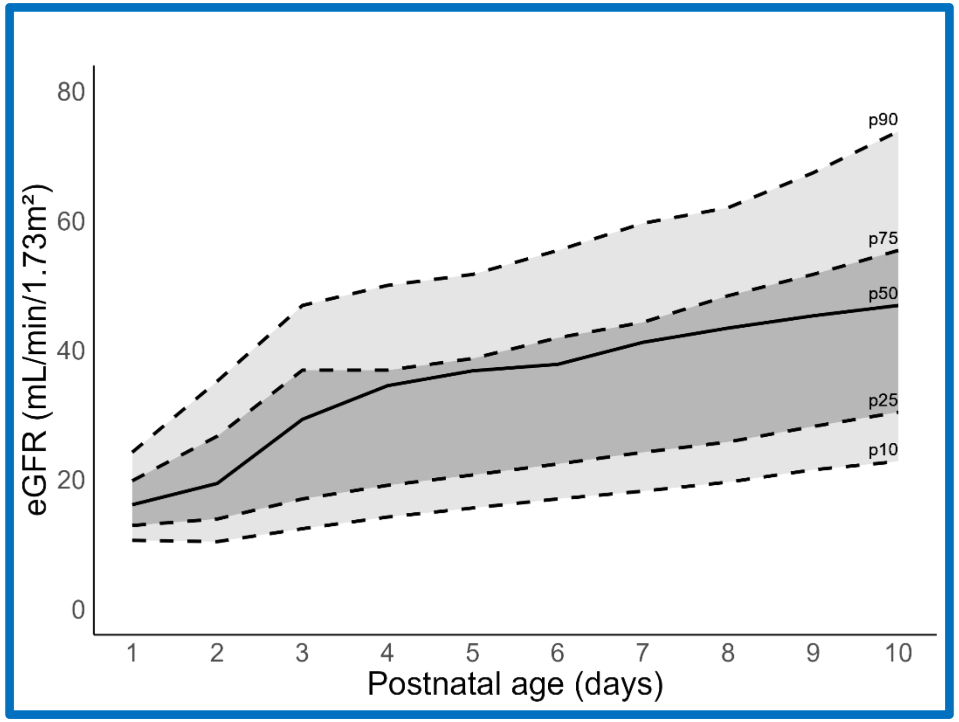

CONCLUSION: eGFR values for the consecutive days were quantified and a polynomial function was described and compared to healthy term neonates. This equation enables precision pharmacotherapy for GFR cleared drugs in this vulnerable population.

Allegaert K et al. 2025

Pediatric Nephrology
Journal of the
IPNA International Pediatric Nephrology Association
